# Rasagiline Pharmacokinetics in CYP1A2 Variant Healthy Smokers & Non-smokers in Different Doses

**DOI:** 10.12669/pjms.38.3.4940

**Published:** 2022

**Authors:** Rabiea Bilal, Naseem Saud Ahmad, Sehrish Zaffar, Muhammad Usama Mazhar, Waqar Ahmed Siddiqui, Saba Tariq

**Affiliations:** 1 Rabiea Bilal- Ph.D Pharmacology, Professor, Combined Military Hospital (CMH) Lahore Medical College & Institute of Dentistry, National University of Medical Sciences (NUMS), Lahore, Pakistan. University of Health Sciences (UHS), Lahore, Pakistan; 2 Naseem Saud Ahmad-Ph.D Pharmacology, Professor, Department of Pharmacology, University of Health Sciences (UHS), Lahore, Pakistan; 3 Sehrish Zaffar- M.Phil Pharmacology, Assistant Professor, Department of Pharmacology, Combined Military Hospital (CMH) Lahore Medical College & Institute of Dentistry, National University of Medical Sciences (NUMS), Lahore, Pakistan; 4 Muhammad Usama Mazhar- M.Phil Pharmacology, Demonstrator, Department of Pharmacology, Combined Military Hospital (CMH) Lahore Medical College & Institute of Dentistry, National University of Medical Sciences (NUMS), Lahore, Pakistan; 5 Waqar Ahmed Siddiqui- M.Phil Pharmacology, Assistant Professor, Department of Pharmacology, Combined Military Hospital (CMH) Lahore Medical College & Institute of Dentistry, National University of Medical Sciences (NUMS), Lahore, Pakistan; 6 Saba Tariq- Ph.D Pharmacology, Professor, Department of Pharmacology, University Medical & Dental College, The University of Faisalabad, Pakistan

**Keywords:** Bioavailability, Drug disposition, Genetic variation, Pharmacokinetics, Smokers

## Abstract

**Objectives:**

Rasagiline, a drug for Parkinson’s disease is metabolized by CYP1A2 enzyme. The objective of the study was to investigate the influence of cytochrome P450 1A2 variants and smoking status of healthy individuals on the pharmacokinetics of rasagiline.

**Methods:**

A comparative, open label, interventional, single oral dose, pharmacokinetic study was performed on 108 healthy volunteers in UHS & UVAS, Lahore. Data collection was initiated in June 2016 and ended in January 2018. It was divided in three phases with 1, 2 and 5mg of rasagiline given to a group of 36 volunteers in each phase. Volunteers were sub-divided into six groups of AA smokers, AA non-smokers, AC smokers, AC non-smokers, CC smokers & CC non-smokers on the basis of genotyping and smoking status. Serial blood sampling was performed at 0, 0.25, 0.5, 0.75, 1, 1.5, 2, 3, 4, 6, 8 & 12 hours after administration of rasagiline tablets. Plasma concentrations were determined using High Performance Liquid Chromatography (HPLC) method. Pharmacokinetic (PK) parameters were calculated using software (APO) pharmacological analysis.

**Results:**

Analysis of variance (ANOVA) showed significant difference between AA and CC groups. Multiple group comparison with post hoc Tukey’s revealed that AA-smokers had significantly less t_max_ (p<0.001), t_1/2_ (p<0.012), AUC (p<0.008) and highest Cl (p<0.001) as compared to CC-smokers. The trend was same across all three doses.

**Conclusion:**

The study concludes that the systemic metabolism of rasagiline is significantly increased in CYP1A2*AA variants while smoking status did not show consistent difference in PK parameters.

***Registered Trial:*** ISRCTN68198254

## INTRODUCTION

Rasagiline is used to treat Parkinson’s disease in dose of 0.5mg or 1mg in combination therapy with carbidopa and levodopa or as monotherapy. It is a monoamine oxidase B inhibitor that undergoes extensive hepatic metabolism by the enzyme cytochrome (CYP) P4501A2. The drug is absorbed from gastrointestinal tract and has half-life of 1.5-3.5 hours according to the dose administered. The oral bioavailability of the drug is 35% and less than 1% of it is excreted unchanged in urine.^1^

Pharmacokinetic studies can be an important tool for adjusting the dose of drugs metabolized by polymorphic genes of drug metabolizing enzymes in different individuals. Clinically significant pharmacokinetic interactions have been reported earlier between genetic polymorphism and drugs like omeprazole, diazepam and clozapine. Some of these have resulted in toxic effects and some in therapeutic failure, depending upon the plasma levels obtained due to genetic variability in population. The mutation 163C>A of CYP1A2 appears to be associated with an increase in enzyme activity.^2^ According to Ensemble genome web browser, in Punjabi population of Lahore (PJL) Pakistan, the prevalence of allele A is 54% and C is 45% for rs762551 with prevalence of A/A 29%, A/C 50% & C/C 21%. It can be postulated that, for a drug whose main route of elimination is metabolism by a particular enzyme, an absolute or relative deficiency of that enzyme will lead to increased plasma drug levels.^3^

The prevalence of smoking in Pakistan is 19.1%.^4^ Cigarette smoking induces the activity of cytochrome P450 (CYP) 1A2 via chemicals in cigarette smoke such as polycyclic aromatic hydrocarbons.^5^ There may be transcriptional activation of CYP1A2 gene.^6^ This may lead to decreased plasma concentration of drug and a reduction in the effectiveness of the substrate drug. Literature reveals that incase a patient stops smoking while on drug therapy, the dose needs to be optimized as the activity of CYP enzyme reverts on cessation of smoking and the drug levels may persist for longer time in plasma.^7^ Our study is a step towards precision medicine. The objective of the study was to investigate the influence of cytochrome P450 1A2 variants and smoking status of healthy individuals on the pharmacokinetics of rasagiline.

## METHODS

### Subjects and Study Protocols

A comparative, open label, interventional, single oral dose, pharmacokinetic study was performed on 108 healthy volunteers in three phases over a period of one year after obtaining written informed consent from them. The study was approved by the Independent Ethics Committee of University of Veterinary and Animal Sciences, Lahore and Ethics Review Committee of University of Health Sciences, Lahore, Pakistan (Best-16 25/8/16 & ISRCTN 68198254). The procedures followed were in accordance with the institutional guidelines that follow recommendations from the Declaration of Helsinki.

Thirty-six healthy volunteers were taken in each of the three phases with 1, 2 & 5mg dose respectively. Inclusion criteria was healthy volunteers of both sexes with age between 18 to 30 years. Volunteers were excluded if they had deranged CBC, LFT, RFT or any unstable medical condition. Those receiving any medication which is inducer or inhibitor or substrate for CYP1A2 were also excluded. They were sub-divided into six groups on the basis of their genetic and smoking status as AA smokers, AA non-smokers, AC smokers, AC non-smokers, CC smokers & CC non-smokers. Participants of first phase were given 1mg tablet of rasagiline (Alzilo^®^ tablet, Searle Pharmaceuticals, Karachi, Pakistan) with 240ml glass of water after an overnight fast of 10 hours. Serial blood sampling of 3ml each was performed at 0, 0.25, 0.5, 0.75, 1, 1.5, 2, 3, 4, 6, 8 & 12 hours where, zero hour refers to pre-drug administered sample. Plasma was separated from whole blood by centrifugation and stored at -20^0^C for drug analysis. Standardized meals were offered to volunteers 3 hours after drug administration. They were monitored by the qualified physicians and nurses throughout the study period. The same process was repeated for 2 & 5mg doses.

Genotyping was performed before the volunteers were divided into groups. Genomic DNA was separated from the whole blood by standard organic method,^8^ and was stored at -80^0^C. The intron 1 region of CYP1A2*1F was region of interest. Amplification was performed using PCR method with forward and reverse primers of corresponding region of gene. PCR based assay are simple and reliable for a use in a Pakistani setting.^9^ The experiment was performed on Thermal Cycler Proflex PCR System (Life technologies, CA). Primers were adjusted according to optimized protocol. Sequencing of PCR product was performed in 96 well Micro Amp PCR plate. The Big Dye Terminator V3.1 cycle sequencing kit was used. The reaction mix was analyzed on ABI 3730 Genetic Analyzer.

Smoking was defined as present if a participant reported to be smoking at the time of survey either daily or occasionally. It was confirmed by performing the urine cotinine dipstick test a day earlier the drug was given for pharmacokinetic part of the study. Non-smoking status was confirmed by the urine dipstick test.^10^

The protein precipitation method as reported by Ravi et al^11^ was used to extract rasagiline from plasma. Six hundred microliter plasma and 900µl acetonitrile were pipetted in eppendroff tube, followed by vortex mixing for two minutes and centrifugation of sample for 20 minutes at 10,500 rpm for 10 minutes at 4^0^C. Supernatant was separated and transferred to a sample loading vial and 80μl was injected into HPLC system (Shimadzu, Germany). Different mobile phase ingredients and ratios were tested and flow rates were adjusted. Finally, the precision and accuracy of quality control samples was determine on the basis of peak properties, convenience of preparation, sensitivity of method and applicability of method to quantify rasagiline in human plasma. A mixture of mobile phase consisted of 10 mM ammonium acetate (NH_4_CH_3_CO_2_) and acetonitrile (55:45, v/v) was adjusted to pH 5.8. The buffer after being filtered through a 0.22μm filter paper was added to acetonitrile and mixed thoroughly. The separation was performed at room temperature on LiChrosphere®RP-18 silica based HPLC column with dimensions of 250 x 4.6 mm, 5 μm particle size (Merck KGaA, Darmstadt, Germany). The mobile phase was pumped at a flow rate of 1 mL/min. Rasagiline was detected with the help of a UV detector at 265 nm wavelength as a peak appearing at seven minutes.

### Statistical analysis

Data analysis was carried out by using statistical packages for social sciences (SPSS) version 20. Mean ± S.D. (Standard Deviation) was calculated for normally distributed quantitative variables of pharmacokinetics. Shapiro-wilk test was chosen to check normality of data and one-way ANOVA was used to find group mean differences. Post hoc Tukey test was applied to determine which groups means differ. For all statistical tests, the ‘value of p’ equal to or less than 0.05 was considered significant. The software used for calculating pharmacokinetic parameters was APO pharmacological analysis (MW/PHARM, version 3.02, Holland).

## RESULTS

Mean age of participants was 24 years and mean body weight was 64.5kg. The frequencies of AA, AC, CC variants were 38%, 43% and 19% respectively. The six pharmacokinetic parameters that were undertaken for this study were C_max_, t_max_, t_1/2_, AUC, Vd and Cl. Post hoc Tukey’s comparison was carried out between six groups AA smokers, AA non-smokers, AC smokers, AC non-smokers, CC smokers & CC non-smokers. It was observed that C_max_ was significantly higher in AA-smokers group as compared to the other five groups and the trend was same across all three doses (1mg, 2mg & 5mg). Similarly, t_max_ was significantly less in AA-smokers as compared to rest of the five groups and results were same for all doses. AA smokers & nonsmokers both groups had significantly less t_1/2_ as compared to AC & CC smokers & nonsmokers. The difference was greatest between AA & CC genetic variants in all three doses. AA-smokers and nonsmokers had significantly less AUC as compared to other groups at all three doses. Volume of distribution did not show any significant pattern. Clearance of rasagiline also showed the same trend across all three doses. It was highest in AA smokers & nonsmokers as compared to AC and CC smokers & nonsmokers. The difference was greatest between AA smokers and CC-nonsmokers at 2 & 5mg doses. Descriptive statistics with mean ± SD for all the measured pharmacokinetic variables across all three doses 1, 2 & 5mg. is shown in Table-I

**Table-I:** Pharmacokinetics of CYP1A2 variants smokers & non-smokers at 1, 2 & 5mg of rasagiline (n=108).

Variable	Groups	Mean ± SD at 1mg	P value	Mean ± SD at 2mg	P value	Mean ± SD at 5mg	P value
C_max (μg/l)_	AA-Smoker	225.2 ± 49.3	<0.001	490.8 ± 26.9	<0.001	1299.7 ± 135.4	<0.001
	AA-Non-smoker	115.1 ± 14.1		349.2 ± 30.7		997.6 ± 35.2	
	AC-Smoker	133.1 ± 17.1		351.1 ± 19.4		933.6 ± 21.7	
	AC-Non-smoker	124.8 ± 8.3		337.5 ± 12.6		906.7 ± 9.0	
	CC-Smoker	111.9 ± 7.2		325.9 ± 61.2		794.0 ± 87.4	
	CC-Non-smoker	119.6 ± 14.4		299.8 ± 31.8		941.9 ± 16.8	
t_max (h)_	AA-Smoker	0.356 ± 0.059	<0.001	0.320 ± 0.005	<0.001	0.455 ± 0.075	<0.001
	AA-Non-smoker	0.728 ± 0.082		0.735 ± 0.025		0.661 ± 0.012	
	AC-Smoker	0.882 ± 0.163		0.887 ± 0.051		0.651 ± 0.018	
	AC-Non-smoker	0.704 ± 0.149		0.805 ± 0.065		0.609 ± 0.028	
	CC-Smoker	0.871 ± 0.044		0.909 ± 0.112		0.721 ± 0.088	
	CC-Non-smoker	0.697 ± 0.108		1.056 ± 0.119		0.963 ± 0.040	
t_1/2 (h)_	AA-Smoker	0.373 ± 0.198	0.001	0.478 ± 0.229	0.002	0.480 ± 0.054	0.002
	AA-Non-smoker	0.473 ± 0.123		0.453 ± .169		0.339 ± 0.009	
	AC-Smoker	1.156 ± 0.309		0.580 ± 0.185		0.881 ± 0.061	
	AC-Non-smoker	1.257 ± 0.733		0.908 ± 0.365		1.190 ± 0.027	
	CC-Smoker	1.347 ± 0.683		0.913 ± 0.348		1.151 ± 0.127	
	CC-Non-smoker	1.165 ± 0.366		1.379 ± 0.180		1.170 ± 0.214	
AUC _(h.μg/l)_	AA-Smoker	167.1 ± 28.1	0.002	445.7 ± 23.9	0.001	1267.3 ± 58.2	0.001
	AA-Non-smoker	194.8 ± 25.7		567.3 ± 87.0		1324.7 ± 41.1	
	AC-Smoker	349.5 ± 55.6		686.9 ± 45.3		1724.2 ± 68.5	
	AC-Non-smoker	309.4 ± 135.2		723.9 ± 92.1		1989.5 ± 63.2	
	CC-Smoker	333.8 ± 84.6		810.5 ± 107.9		1894.2 ± 65.3	
	CC-Non-smoker	286.7 ± 71.6		934.5 ± 185.4		2658.2 ± 139.8	
Vd _(l)_	AA-Smoker	0.0032 ± 0.0018	0.012	0.0031 ± 0.0015	0.012	0.0027 ± 0.0002	0.012
	AA-Non-smoker	0.0035 ± 0.0008		0.0023 ± 0.0004		0.0018 ± 0.0001	
	AC-Smoker	0.0049 ± 0.0017		0.0024 ± 0.0006		0.0037 ± 0.0001	
	AC-Non-smoker	0.0056 ± 0.0009		0.0035 ± 0.0011		0.0043 ± 0.0001	
	CC-Smoker	0.0056 ± 0.0023		0.0033 ± 0.0014		0.0044 ± 0.0004	
	CC-Non-smoker	0.0058 ± 0.0005		0.0043 ± 0.0006		0.0032 ± 0.0004	
CL _(l/h)_	AA-Smoker	0.0061 ± 0.0012	<0.001	0.0045 ± 0.0002	<0.001	0.0040 ± 0.0002	<0.001
	AA-Non-smoker	0.0052 ± 0.0007		0.0036 ± 0.0005		0.0038 ± 0.0001	
	AC-Smoker	0.0029 ± 0.0004		0.0029 ± 0.0002		0.0029 ± 0.0001	
	AC-Non-smoker	0.0037 ± 0.0012		0.0028 ± 0.0004		0.0025 ± 0.0001	
	CC-Smoker	0.0032 ± 0.0008		0.0025 ± 0.0003		0.0027 ± 0.0001	
	CC-Non-smoker	0.0037 ± 0.0008		0.0022 ± 0.0004		0.0019 ± 0.0001	

## DISCUSSION

In the present research work, we studied the influence of pharmacogenetics and smoking over pharmacokinetics of rasagiline. To the best of our knowledge, no such work has been done before. It is an important step towards precision medicine.

The results of our study were intriguing, AA smokers had significantly different PK parameters as compared to rest of the five groups. The trend was almost same in all three doses given as shown in Table-I, suggesting that interindividual differences in AUC, t_1/2_ and Cl were mainly responsible for the pharmacokinetic variation of rasagiline. Keeping in view all intergroup comparisons, there was a stronger influence of genetic variation as compared to smoking status on rasagiline pharmacokinetics. The results were consistent with Woo HI et al^12^ who found genetic association with pharmacokinetics of atorvastatin and findings of Azam F et al identifying the role of ABCB1 polymorphism in achieving optimal levels of tacrolimus during dose titration.^13^

CYP enzymes alter pharmacokinetics of drugs. Javed A reported side effects of chloroquine when given in combination with CYP3A4 inhibitors.^14^ The enzyme CYP1A2 has 36 allelic variants. It is highly inducible both in terms of enzyme activity and its genetic expression.^15^ Allele A of CYP1A2 enzyme had been reported to have higher enzyme inducing activity in smokers.^16^ Genetic polymorphism of metabolizing enzymes have shown association with oral cancers.^17^

C_max_ is the maximum plasma concentration of a drug and it is dependent on rate & extent of drug absorption in plasma.^18^ It was highest in the fast metabolizer group of our study. The results were consistent with the work of Ahmed et al on Omeprazole PK in Pakistani population.^19^

The AUC calculated by Trapezoidal rule^20^ representing bioavailability of drug was almost half in AA-smokers as compared to the CC-smokers. The reason could be fast metabolism of drug in plasma due to higher enzyme activity. It was further evident by shortest half-life in AA-smokers group. Where the values were one-third as compared to CC-smokers & non-smokers. These were consistent with the results of Blake CM et al^21^ who presented analysis on pharmacokinetics of metaprolol and with Woo^13^ who reported influence of genetic polymorphism on pharmacokinetics of drugs. The largest clearance observed in AA smokers group further augments the proposal of making a special strategy for dosing of rasagiline in this particular group. The study done by Ahmed et al suggests that pharmacogenetics screening for low activity genotypes would be a helpful tool for clinicians when they prescribe medications metabolized by CYP enzymes.^22^ Our study results are consistent with the work done by Qayyum A et al on warfarin plasma levels, influenced by CYP2C9 variants in Pakistani population.^23^

The co-administration of Carbidopa with Levodopa in the past for treatment of Parkinson’s is a proof that a change in treatment modality is essential to improve bioavailability, half-life and clearance of a drug to keep its therapeutic effectiveness intact. Also, the factors influencing pharmacokinetics of drugs should be taken into account for desired therapeutic effect.

### Limitation

Data should have been collected from Parkinson’s patients but due to old age and multiple drug therapies, it was not possible in our limited resources.

## CONCLUSION

Taken together, we conclude that the difference in PK between slow (CC) and fast (AA) metabolizers was significant in all parameters. The drug rasagiline may need dose adjustment in fast metabolizers to attain the therapeutic levels. However, the effect of smoking status was not significant at this dose. Further studies should be undertaken on patients taking rasagiline to check if genetic variation can have a true clinical impact.

### Authors’ Contribution:

**RB:** Responsible and accountable for the accuracy or integrity of the work. Designing of research project and acquisition, analysis & interpretation of data

**NSA:** Supervising the research, conception & designing of work with critical intellectual drafting.

**SZ:** Literature search, interpretation of genetic analysis and manuscript writing

**MUM:** Data collection, interpretation of data & manuscript editing.

**WAS:** Data collection, Interpretation of data and drafting of work

**ST:** Acquisition & interpretation of data, critical revision & editing of final manuscript.
